# Correction: Interpretable multimodal classification for age-related macular degeneration diagnosis

**DOI:** 10.1371/journal.pone.0329294

**Published:** 2025-07-29

**Authors:** The PLOS One Staff

There is an error in affiliation 1 for author Carla Vairetti. The correct affiliation 1 is: Facultad de Ingeniería y Ciencias Aplicadas, Universidad de los Andes, Santiago, Chile.

The publisher apologizes for the error.
