# Supplementary material for: Interpretable multimodal classification for age-related macular degeneration diagnosis
Source: PLoS One. 2024 Nov 11;19(11):e0311811. doi: 10.1371/journal.pone.0311811 (PMC11554086; doi:10.1371/journal.pone.0311811)
Supplement: Attachment — Submitted filename: Response to reviewers Plos One R1.docx [file pone.0311811.s001.docx]

**Point-to-Point responses to the Editors and Reviewers**

| **Editor** | |
| --- | --- |
| **General Comment** | Journal requirements:  When submitting your revision, we need you to address these additional requirements.  1. Please ensure that your manuscript meets PLOS ONE's style requirements, including those for file naming. The PLOS ONE style templates can be found at <https://journals.plos.org/plosone/s/file?id=wjVg/PLOSOne_formatting_sample_main_body.pdf> and <https://journals.plos.org/plosone/s/file?id=ba62/PLOSOne_formatting_sample_title_authors_affiliations.pdf>  2. Please note that PLOS ONE has specific guidelines on code sharing for submissions in which author-generated code underpins the findings in the manuscript. In these cases, all author-generated code must be made available without restrictions upon publication of the work. Please review our guidelines at <https://journals.plos.org/plosone/s/materials-and-software-sharing#loc-sharing-code> and ensure that your code is shared in a way that follows best practice and facilitates reproducibility and reuse.  3. Thank you for stating the following financial disclosure:   [The authors gratefully acknowledge financial support from ANID PIA/PUENTE AFB230002 and FONDECYT-Chile, grants 12200007, 1200221, 11191215, and 1212038.].   Please state what role the funders took in the study.  If the funders had no role, please state: "The funders had no role in study design, data collection and analysis, decision to publish, or preparation of the manuscript."  If this statement is not correct you must amend it as needed.  Please include this amended Role of Funder statement in your cover letter; we will change the online submission form on your behalf.  4. In the online submission form, you indicated that [Data cannot be shared publicly because it belongs to the Hospital Clínico Universidad de Chile, Santiago, Chile. The data can be shared privetely upon reasonable request.].  All PLOS journals now require all data underlying the findings described in their manuscript to be freely available to other researchers, either 1. In a public repository, 2. Within the manuscript itself, or 3. Uploaded as supplementary information. This policy applies to all data except where public deposition would breach compliance with the protocol approved by your research ethics board. If your data cannot be made publicly available for ethical or legal reasons (e.g., public availability would compromise patient privacy), please explain your reasons on resubmission and your exemption request will be escalated for approval.  5. We note that Figure 3,4,5,6,7,8, and 9 in your submission contain [map/satellite] images which may be copyrighted. All PLOS content is published under the Creative Commons Attribution License (CC BY 4.0), which means that the manuscript, images, and Supporting Information files will be freely available online, and any third party is permitted to access, download, copy, distribute, and use these materials in any way, even commercially, with proper attribution. For these reasons, we cannot publish previously copyrighted maps or satellite images created using proprietary data, such as Google software (Google Maps, Street View, and Earth). For more information, see our copyright guidelines: <http://journals.plos.org/plosone/s/licenses-and-copyright>.   We require you to either (1) present written permission from the copyright holder to publish these figures specifically under the CC BY 4.0 license, or (2) remove the figures from your submission:   - You may seek permission from the original copyright holder of Figure 3,4,5,6,7,8, and 9   to publish the content specifically under the CC BY 4.0 license.     We recommend that you contact the original copyright holder with the Content Permission Form (<http://journals.plos.org/plosone/s/file?id=7c09/content-permission-form.pdf>) and the following text: “I request permission for the open-access journal PLOS ONE to publish XXX under the Creative Commons Attribution License (CCAL) CC BY 4.0 (<http://creativecommons.org/licenses/by/4.0/>). Please be aware that this license allows unrestricted use and distribution, even commercially, by third parties. Please reply and provide explicit written permission to publish XXX under a CC BY license and complete the attached form.”   Please upload the completed Content Permission Form or other proof of granted permissions as an ""Other"" file with your submission.   In the figure caption of the copyrighted figure, please include the following text: “Reprinted from [ref] under a CC BY license, with permission from [name of publisher], original copyright [original copyright year].”   - If you are unable to obtain permission from the original copyright holder to publish these figures under the CC BY 4.0 license or if the copyright holder’s requirements are incompatible with the CC BY 4.0 license, please either i) remove the figure or ii) supply a replacement figure that complies with the CC BY 4.0 license. Please check copyright information on all replacement figures and update the figure caption with source information. If applicable, please specify in the figure caption text when a figure is similar but not identical to the original image and is therefore for illustrative purposes only. The following resources for replacing copyrighted map figures may be helpful:   USGS National Map Viewer (public domain): <http://viewer.nationalmap.gov/viewer/> The Gateway to Astronaut Photography of Earth (public domain): <http://eol.jsc.nasa.gov/sseop/clickmap/> Maps at the CIA (public domain): <https://www.cia.gov/library/publications/the-world-factbook/index.html> and <https://www.cia.gov/library/publications/cia-maps-publications/index.html> NASA Earth Observatory (public domain): <http://earthobservatory.nasa.gov/> Landsat: <http://landsat.visibleearth.nasa.gov/> USGS EROS (Earth Resources Observatory and Science (EROS) Center) (public domain): [http://eros.usgs.gov/#](http://eros.usgs.gov/) Natural Earth (public domain): <http://www.naturalearthdata.com/>  Additional Editor Comments:  The authors need to address the reviewer's comments carefully. It is also recommended to cite the few relevant recent studies of current year 2024. |
| **Author Answer** | Thank you very much for your valuable feedback. Below, we respond to each of your questions:   1. We have adjusted the format of the figures, numbering them from Fig1 to Fig30. We have also provided the .tiff files for each of the figures. Please note that the entire manuscript is formatted in LaTeX using the PLOS One template. 2. We agree with the statement. The code will be uploaded to a public repository following the journal’s guidelines in case of publication. 3. The funders mentioned in the original submission had no role in study design, data collection and analysis, decision to publish, or preparation of the manuscript. We have adjusted the acknowledgments accordingly. 4. Our data cannot be made publicly available for ethical and legal reasons. Public availability would compromise patient privacy, and public deposition would breach compliance with the protocol approved by our research ethics board (the original files and their translations were attached in the previous submission). Therefore, we request an exemption for this study in this cover letter. 5. This submission does not include map/satellite images or any copyrighted material. All the images are either diagrams designed for this study or OCT images collected by our research team in accordance with the agreements with the Ethics Board.   Regarding the final comment:   1. We have carefully addressed all the reviewers' comments, which are highly appreciated. 2. Recent references have been included in the revised version of the manuscript. |

| **Reviewer #1** | |
| --- | --- |
| **Comment 1.1** | • Novelty of combining optical coherence tomography (OCT) and infrared reflectance (IR) imaging is mentioned, but the paper does not sufficiently compare this with existing methods or justify the advancements over prior approaches.  • Provide a comparative analysis of your method with recent studies in multimodal imaging for AMD and explain specifically how your approach advances the field. Include relevant recent literature to strengthen the argument. |
| **Author Answer** | Thank you very much for your valuable feedback. Next, we respond each one of your questions. |
| **Author Action** | *We have extended the discussion on multimodal imaging for AMD diagnosis, highlighting the novelty and contribution of our study in relation to recent studies.*  *The following paragraphs were adjusted/included in Section “Theoretical background on multimodal OCT and XAI techniques”:*  *Several papers have proposed a multimodal approach to AMD detection. Among the seminal studies, Vaghefi et al. [14] combined OCT and CFP imaging using Inception-ResNet-v2. Alternatively, Yoo et al. [15] also combined OCT and fundus imaging using restricted Boltzmann machine (RBM) and deep belief network (DBN). Although both studies conclude that the combination of different sources is better than a single image, their main issue is the sample size: n=75 and n=83 for Vaghefi et al. [14] and Yoo et al. [15], respectively. A larger study (n=2450) was designed by Keenan et al. [3], which combined FAF and CFP to detect reticular pseudodrusen (RPD). This approach, however, combines images of a similar nature (fundus imaging), excluding OCT samples from the analysis.*  *In recent years, research on multimodal AMD diagnosis has focused on the analysis of different imaging modalities and AMD variants, such as dry AMD [8]. Wang et al. [16] presented a multimodal approach to AMD detection using OCT and CFP imaging. Anegondi et al. [6] utilized multimodal deep learning to predict the annualized growth rate of geographic atrophy (GA) using OCT and FAF imaging. GA was also analyzed by Winkler et al. [2] using FAF and fundus photos (FP). Oncel et al. [5] examined the relationship between intraretinal hyperreflective foci (IHRF) identified on optical OCT B-scans and hyperpigmentation on CFP. Finally, Goh et al. [1] showed the benefits of multimodal imaging over CFP alone by combining multiple sources such as OCT, FAF, NIR, and CFP.”*  *We have also improved the discussion on multimodal AMD diagnosis in the introductory section.* |

| **Comment 1.2** | • The introduction is verbose and could be more concise.  • Streamline the introduction to focus on the key points: the clinical importance of AMD, challenges in diagnosis, and how your approach addresses these challenges. Avoid redundant explanations and ensure a clear, direct narrative. |
| --- | --- |
| **Author Answer** | We agree. Thank you for this suggestion. |
| **Author Action** | *We have adjusted the introduction to present a clear and concise motivation for the manuscript, highlighting the main challenges in AMD diagnosis and providing a clear novelty and contribution statement.*  *We have also updated the references on multimodal AMD diagnosis in both the introduction and the prior work section, including four new studies from 2024.* |

| **Comment 1.3** | • The datasets used are not described in enough detail.  • Provide comprehensive details about the datasets, including the number of images, the source, inclusion/exclusion criteria, and any preprocessing steps. Discuss the representativeness of the datasets and potential biases. |
| --- | --- |
| **Author Answer** | Thank you for this remark. We have provided all the information requested by you. |
| **Author Action** | *We have included additional information on the dataset, discussing* *the number of images, the source, inclusion/exclusion criteria, and any preprocessing steps. Discuss the representativeness of the datasets and potential biases.*  *The following paragraphs were adjusted/included in Section “The dataset and experimental setting”:*  *We collected 4563 images of patients seen at the Hospital Clínico Universidad de Chile in Santiago, Chile. This dataset was obtained by scholars and practitioners of the Department of Ophthalmology of the University of Chile.* ***[number of images and source].***  *Each record consists in the combination of an IR and an OCT image extracted from across the macula cube area. The device used to acquire the images corresponds to a Spectralis OCT 2 imaging platform (Heidelberg Engineering, Germany).* ***[source]***  *Each scan was manually labeled as `AMD' (dry AMD or wet AMD), `NORMAL', `OTHER', `UNDEFINED' by two experienced retinal specialists with proper training and clinical experience. If the annotations from the two specialists did not coincide, the images were categorized as `UNDEFINED' and subsequently excluded from the analysis. Additionally, images that presented alterations that may prevent the correct diagnostic interpretation by a trained specialist were also labeled as `UNDEFINED'. These artifacts may be due to errors in the software (misidentification of retinal layers, mirror artifact, cut edge artifact), by the operator (degraded image scan, out of register artifact, off center artifact) or by the patient (motion artifact, off center artifact, degraded image scan, mirror artifact)* [*30, 31]. On the other hand, OCT images with a signal strength index (SSI) lower than 7 were excluded from analysis.* ***[pre-processing and exclusion criteria]***  *The category `OTHER' represents findings that may be found in diseases other than AMD [32], such as: alterations related to vitreo-retinal interface (vitreo-macular traction, lamellar and full-thickness macular hole, epiretinal membrane), suggestive signs of macular edema (intraretinal hyporeflective spaces) or vascular exudation (intraretinal hipereflective dots), retinal/RPE detachments and alterations in normal macular structure (pathologic myopia).* ***[pre-processing and exclusion criteria]***  *Records labeled as `OTHER’ or `UNDEFINED’ were discarded, totaling 237, resulting in a final dataset comprised of 4326 images. These exclusions enable the machine to learn from a more refined dataset, thereby reducing model noise. It is important to note that OCT/Fundus images were selected from patients with a potential AMD diagnosis and do not represent all patients with retinal pathologies treated at the Hospital. Images from healthy controls were individually selected to match the age and sex of each of the patients with potential AMD. However, this process was carried out before applying the exclusion criteria, leading to a greater number of normal cases than AMD samples in the final dataset.* ***[pre-processing and exclusion criteria]***  *The dataset includes 1948 image records with AMD. Within these cases are alterations related to exudative and non-exudative AMD, such as CNV, drusen, and subretinal fluid. The remaining 2378 image records are associated with healthy patients. The images correspond to the eyes of patients with a possible AMD diagnosis, where the retina specialist has made an exhaustive clinical evaluation for the eye or eyes under study. If the patient has findings of possible AMD in both eyes, images from both eyes were included.* ***[pre-processing and additional information]***  *The images were collected retrospectively from medical records between June 1st, 2019, and June 30th, 2021. Only the researchers, students, and practitioners who were involved in collecting the images had access to information that could identify the participants. In the final dataset, all records were handled anonymously.* ***[source and additional information]*** |

| **Comment 1.4** | • The training and validation process of the models lacks detail.  • Elaborate on the model training process, including data partitioning (e.g., train/test split), any cross-validation techniques used, hyperparameter tuning, and criteria for model selection. Include a flowchart or pseudocode if possible. |
| --- | --- |
| **Author Answer** | Thank you for this suggestion. The flowchart of the whole process is presented in Figure 2, including the validation and model selection step. |
| **Author Action** | *The following paragraphs were adjusted/included in Section “The dataset and experimental setting”:*  *The model evaluation was performed as follows: a nested validation strategy was made, splitting the data into a training set (70% of the samples) and a test set (30% of the samples). Ten-fold cross-validation was performed in the training set in order to define the number of epochs and to obtain an average performance. For each set of experiments given by the different combination of data sources (OCT/IR only and the combinations), the models were trained for an increasing number of epochs (from 1 to 15). The best validation performance, computed as the average for all the CNN classifiers, provides the optimal number of epochs for a given set of experiments. Notice that all classifiers have the same number of epochs within a set of experiments.*  *In terms of the model parameters, we considered a batch size of 16, with images of size 248 x 632 pixels. We used the well-known Adam optimizer, with initial learning rates of 0.0001 and 0.00001 for pre-training and fine-tuning, respectively, and β_1_=0.9 and β_2_=0.999. The default configurations were considered for the additional parameters of each CNN classifier. As a standard binary classification problem, cross-entropy was considered as the loss function, and the Rectified Linear Unit (ReLU) as the activation function.*  *In order to make the training process more efficient, we used a `warm start’ procedure, in which the weights obtained in a previous iteration are used as a starting point for the network in the following iteration [33]. All the experiments were performed in Python using the scikit-learn and Keras libraries. The codes can be obtained from the authors upon request. Unfortunately, the dataset cannot be shared due to the rules of confidentiality regarding patient information.*  *The flowchart of this process is presented in Figure 2 including the visualization step in which Grad-CAM and Guided Grad-CAM are considered.* |

| **Comment 1.5** | • The use of Grad-CAM, guided backpropagation, and guided Grad-CAM is described but could be clearer.  • Provide a more detailed explanation of how these techniques work and their implementation in your model. Include diagrams or visual examples to illustrate how these techniques contribute to model interpretability. |
| --- | --- |
| **Author Answer** | Thank you for this suggestion. The flowchart of the whole learning process is presented in Figure 1, including the representation of the XAI techniques. |
| **Author Action** | *The following paragraphs were adjusted/included in Section “Proposed framework for AMD detection using multimodal XAI”:*   - *Grad-CAM represents an evolution of the CAM approach. Grad-CAM takes the gradients of the target object into account to produce a coarse heat-map, emphasizing on the regions of the image that are relevant to distinguish the concept (retinal damage in our case) from other objects. The use of gradients confers versatility and accuracy to the approach [29]. Formally, Grad-CAM computes the gradients of y^c^, the score for class c in {Normal,AMD}, with respect to the activations A_k_ of a convolutional layer, for a given feature map k. The scores are the outputs of the model that result from applying the decision function to each data point, and can be cast into probabilities using the softmax function.* - *Guided backpropagation makes modifications to the raw gradients to provide a visual explanation of a CNN. In contrast to Grad-CAM, it is not class-discriminative in the sense that it generates almost similar visualizations for a given image regardless of the target concept [29]. The output of the model highlights the contour of the objects instead of providing a heat-map, resulting in an interesting alternative to Grad-CAM. This is done by visualizing the positive gradients with respect to the image, suppressing the negative ones when back-propagating to the activation layers.* - *Guided Grad-CAM combines the two previous methods, capturing the best of the two worlds: it is a class-discriminative approach as it is able to locate relevant regions of the target concept, while generating a fine-grained output that defines the contours of it. This is achieved by fusing the two visualizations using element-wise multiplication [29]-*   *The proposed framework is summarized in Figure 1. After cropping and resizing the input image, the corresponding regions are processed by two CNNs, the OCT and IR models. In both networks, we consider a transfer learning approach based on ImageNet. The prediction layer performs the late fusion, combining the two networks to provide a single outcome (either normal or AMD). The output of the proposed framework is twofold: first, the diagnosis is generated for a new image, while a visual justification is also presented by considering three XAI techniques. Based on our results, we propose using two XAI strategies only: Grad-CAM and Guided Grad-CAM. This is discussed in the last section of the manuscript.* |

| **Comment 1.6** | • Results are primarily reported in terms of accuracy.  • Include additional performance metrics such as precision, recall, F1-score, and AUC-ROC to provide a more comprehensive evaluation of your model's performance. Present these in a table for clarity and comparison. |
| --- | --- |
| **Author Answer** | In addition to accuracy, we provide the results in terms of AUC-ROC, which allows for a more comprehensive analysis. For each metric, the standard deviation is also presented. We have also complemented the study with the Friedman and Holm tests for both performance metrics (accuracy and AUC-ROC; see our response to Comment 1.7). |
| **Author Action** | *The following paragraphs were adjusted/included in Section “Proposed framework for AMD detection using multimodal XAI”:*  *Before applying the XAI techniques, we select the best classification strategy using the Area Under the ROC Curve (AUC-ROC) as performance metric on a test set, which remains unseen during the model calibration process. This decision includes the unimodal or multimodal strategies and the CNN architecture.*  *The AUC-ROC represents the area under the receiver operating characteristic (ROC) curve, which plots the true positive rate (TPR) against the false positive rate (FPR) across all possible threshold settings in the training set. Unlike metrics such as accuracy or F1, AUC does not necessitate the selection of a classification threshold. Thus, the best model can be identified using this metric, and subsequently, the threshold can be adjusted based on desired TPR and FPR values. This metric also ensures a balanced accuracy between classes.*  *The following paragraphs were adjusted/included in Section “Classification performance summary”:*  *The performance of the various unimodal and multimodal strategies and CNN classifiers is summarized in Table 1. For each approach, the average AUC-ROC/accuracy of the cross-validation strategy and the test performance for the holdout step are reported. The best test performance is highlighted in bold.* |

| **Comment 1.7** | • The results lack detailed statistical analysis.  • Conduct statistical tests to determine the significance of your findings. Report confidence intervals and p-values where appropriate. Discuss the variability and robustness of your results. |
| --- | --- |
| **Author Answer** | Done. |
| **Author Action** | *The Friedman and Holm tests were applied to analyze whether there are significant differences between the performance of the various CNN classifiers. The p-values for both tests and performance metrics (accuracy and AUC-ROC) are reported. Additionally, the standard deviation is provided for each metric, which, when added to and subtracted from the average performance, leads to the confidence intervals.*  *The following paragraphs were adjusted/included in Section “Classification performance summary”:*  *To determine statistical significance, we applied the Friedman test with the Iman-Davenport correction, as well as the Holm test, both of which are widely used for comparing different machine learning methods [34]. The Friedman test computes a chi-squared statistic to assess whether significant differences exist among the four approaches (OCT only, IR only, uni. OCT+IR, and multi. OCT+IR). The test yielded a value of 3.893, with a p-value below 0.023, allowing us to reject the null hypothesis of similar performances.*  *The Holm test conducts pairwise comparisons between the top-performing approach and the others using Nemenyi's Z test [34]. Table 2 presents the p-value of each test, the significance threshold α_z_/(k-1) (with k={2,3,4} and α_z_=0.05), and the test outcomes in the fifth, sixth, and seventh columns, respectively. A 'reject' outcome is indicated when the p-value falls below the corresponding threshold.*  *[new table: Table 2]*  *In Table 2, we observe that the multimodal approach combining OCT and IR imaging achieves the best average performance across various CNN classifiers, as indicated by the highest AUC-ROC and accuracy values (third and fourth columns, respectively) and the best overall ranking (lowest value in the fifth column). Although this approach does not statistically outperform all the remaining methods (it only outperforms OCT as a single source), we conclude that an average rank of 1.438 across four techniques is a promising result for the proposed framework.* |

| **Comment 1.8** | • The interpretability results are not validated with real-world feedback.  • Consider conducting user studies or obtaining feedback from ophthalmologists to validate the interpretability of the explanations provided by your model. Include their perspectives in the discussion. |
| --- | --- |
| **Author Answer** | It is important to notice that all the interpretations were made by the experienced ophthalmologists in the research team. Prof. Cristhian A. Urzua is an Ophthalmologist (M.D.), specialist in Uveitis and Medical Retina. Graduated from the School of Medicine of the Pontificia Universidad Católica de Chile (Santiago, Chile), he completed his training in Ophthalmology and Immunology at the University of Chile (Santiago, Chile). His research and clinical training in Uveitis and Retina include a Postdoctoral Fellowship at the National Institutes of Health (NIH) in the United States, under the mentorship of Dr. Robert B. Nussenblatt, and two Clinical Fellowships in United Kingdom at Bristol Eye Hospital and Moorfields Eye Hospital, respectively. He is an active member of the International Uveitis Study Group (IUSG) and founder of the Research Network in Ocular Immunology of Latinomerica. Currently, he leads a growing research group in intraocular inflammatory diseases, being the Director of the Laboratory of Autoimmune Ocular Diseases and Associate Professor of Ophthalmology at the University of Chile. His areas of interest include ocular immunology, specifically the development of biomarkers, and clinical research in uveitis and retina.  Notice that the ground-truth in the XAI analysis (red squares in Figures 3a,5a,6a,7a,8a, and 9a) was also defined by Prof. Urzua. We agree that a explanatory note is required. |
| **Author Action** | *The following paragraphs were adjusted/included in Section “Results for the XAI methods and discussion”:*  *In the following figures, we discuss the visual justifications for the best classifier, which is the proposed multimodal strategy with the OCT and IR images as inputs, and ResNet as CNN classifier. Each figure presents four images: the input image, and the visualization of the three XAI methods: Grad-CAM, Guided Backpropagation, and Guided Grad-CAM. his analysis was designed and validated by Prof. C. Urzua, an experienced ophthalmologist and co-author of the paper. He also defined the ground truth (represented by red squares in the figures) and the regions of interest in the images.* |

| **Comment 1.9** | • The discussion lacks depth regarding practical implications.  • Discuss how your system could be integrated into clinical practice, potential challenges in deployment, and how it might impact clinical workflows. Address any limitations and suggest ways to overcome them. |
| --- | --- |
| **Author Answer** | Thank you for these suggestions. |
| **Author Action** | *We have improved the discussion on the practical implications and limitations; see Section “Conclusions”. The following paragraphs were included/adjusted:*  *In terms of practical implications, the proposed system can be integrated into clinical practice to perform automatic analysis of OCT/IR imaging. The system can provide insights that may be difficult for ophthalmologists to identify on their own. To ensure successful integration, it is essential to adopt a collaborative approach where AI tools augment, rather than replace, the expertise of healthcare professionals. This requires proper training, ongoing evaluation of AI performance, and a robust infrastructure to support the seamless incorporation of AI into existing clinical workflows.*  *The main limitation of the framework regarding its integration into clinical practice is that it generates four new images based on XAI methods: the OCT and IR images along with the outcomes of the Grad-CAM and Guided Grad-CAM methods. This may result in an overwhelming amount of information for the ophthalmologist, even with the removal of Guided Backpropagation. As future work, we intend to enhance this framework by ranking the XAI images based on their ability to aid in diagnosis through an automated process. For a given patient, the optimal combination of visual justifications might be the Grad-CAM output for the IR image and the Guided Grad-CAM output for the OCT image, for example, while different combinations of images could be more suitable in other cases.* |

| **Comment 1.10** | • Figures and tables could be improved to better illustrate key points.  • Ensure all figures and tables are well-labeled and add explanatory captions. Use visual examples to show how the interpretability techniques work on specific cases of AMD. Consider adding a figure that summarizes the workflow of your method. |
| --- | --- |
| **Author Answer** | Thank you for these suggestions. |
| **Author Action** | *The revised manuscript includes two flowcharts that describe the method's functioning: Figure 1 presents a diagram for multimodal AMD prediction using CNNs and XAI techniques, while Figure 2 shows a flowchart of the four-step framework followed in the experimental section. Additionally, the manuscript now includes six illustrative examples demonstrating how the XAI techniques work on specific AMD cases. The captions have been revised to improve clarity.* |

| **Comment 1.11** | • The conclusion does not clearly summarize the specific contributions and implications.  • Provide a more definitive conclusion that summarizes the key contributions of your work, its impact on the field, and specific advancements over prior methods. Outline clear directions for future research and how your findings can be further validated and applied. |
| --- | --- |
| **Author Answer** | Thank you for this suggestion. |
| **Author Action** | *The following paragraph was included/adjusted in Section “Conclusions”.*  *Our contribution is mainly applied as this is the first XAI study that combines OCT and IR imaging in a multimodal fashion. We show empirically the importance of IR imaging since it provides a general overview of the macula, complementing the information of the OCT image. We can observe in the IR image subretinal alterations in the retinal photoreceptor layer, the choroid, and the retinal pigmented epithelium (RPE) [36].*  *In terms of practical implications, the proposed system can be integrated into clinical practice to perform automatic analysis of OCT/IR imaging. The system can provide insights that may be difficult for ophthalmologists to identify on their own. To ensure successful integration, it is essential to adopt a collaborative approach where AI tools augment, rather than replace, the expertise of healthcare professionals. This requires proper training, ongoing evaluation of AI performance, and a robust infrastructure to support the seamless incorporation of AI into existing clinical workflows.*  *The main limitation of the framework regarding its integration into clinical practice is that it generates four new images based on XAI methods: the OCT and IR images along with the outcomes of the Grad-CAM and Guided Grad-CAM methods. This may result in an overwhelming amount of information for the ophthalmologist, even with the removal of Guided Backpropagation. As future work, we intend to enhance this framework by ranking the XAI images based on their ability to aid in diagnosis through an automated process. For a given patient, the optimal combination of visual justifications might be the Grad-CAM output for the IR image and the Guided Grad-CAM output for the OCT image, for example, while different combinations of images could be more suitable in other cases.* |

| **Comment 1.12** | • There are instances of verbose language and potential typos.  • Conduct a thorough proofreading to correct any typos and improve language clarity. Consider using more concise phrasing where possible to enhance readability. |
| --- | --- |
| **Author Answer** | Thank you very much for this suggestion. |
| **Author Action** | *The entire manuscript was reviewed by a native speaker to improve clarity and eliminate any typos or grammatical errors. The revised version of the manuscript is now a more concise version of the text.* |

| **Comment 1.13** | • The references section may not include the latest relevant research.  • Update the references to include recent literature relevant to your study, particularly in the fields of XAI, multimodal imaging, and AMD diagnosis. Ensure all citations are current and properly formatted. |
| --- | --- |
| **Author Answer** | Thank you very much for this suggestion. |
| **Author Action** | *Recent references on XAI, multimodal imaging, and AMD diagnosis have been included in the revised version of the manuscript.* |

| **Reviewer #2** | |
| --- | --- |
| **General Comment** | I believe this is a very good study with the potential to help improve the use of AI in diagnosis of retinal diseases. The authors have done a good job with a fairly large sample size, they have also highlighted the strengths and weaknesses of the study and also areas for further research. |
| **Author Answer** | Thank you very much for your positive comments and encouraging feedback. |
